# Relationship of the Cold-Heat Sensation of the Limbs and Abdomen with Physiological Biomarkers

**DOI:** 10.1155/2016/2718051

**Published:** 2016-10-12

**Authors:** Duong Duc Pham, JeongHoon Lee, GaYul Kim, JiYeon Song, JiEun Kim, Chae Hun Leem

**Affiliations:** Department of Physiology, Ulsan College of Medicine, 388-1 Poongnap-dong, Songpa-gu, Seoul, Republic of Korea

## Abstract

The present study explored the relationship between the regional Cold-Heat sensation, the key indicator of the Cold-Heat patterns in traditional East Asian medicine (TEAM), and various biomarkers in Korean population. 734 apparently healthy volunteers aged 20 years and older were enrolled. Three scale self-report questions on the general thermal feel in hands, legs, and abdomen were examined. We found that 65% of women tended to perceive their body, particularly their hands and legs, to be cold, versus 25% of men. Energy expenditure and temperature load at resting state were lower in women, independently of body mass index (BMI). Those with warm hands and warm legs had a 0.74 and 0.52 kg/m^2^ higher BMI than those with cold hands and cold legs, respectively, regardless of age, gender, and body weight. Norepinephrine was higher, whereas the dynamic changes in glucose and insulin during an oral glucose tolerance test were lower in those with cold extremities, particularly hands. No consistent differences in biomarkers were found for the abdominal dimension. These results suggest that gender, BMI, the sympathetic nervous system, and glucose metabolism are potential determinants of the Cold-Heat sensation in the hands and legs, but not the abdomen.

## 1. Introduction

Traditional East Asian medicine (TEAM) refers to various forms of traditional medicine mostly practiced in East Asian countries, such as traditional Chinese medicine, Kampo medicine (traditional Japanese medicine), traditional Korean medicine, and traditional Vietnamese medicine [1]. In TEAM, pattern identification (PI), a diagnostic method based on holistic evaluations of clinical symptoms used to define the cause, location, and nature of illness, plays a key role in all aspects of TEAM. The four main patterns are Yin-Yang, Cold-Heat, Deficiency-Excess, and Interior-Exterior [2]. Although there have been several attempts to explore the essence of PI using scientific principles [3–6], no consensus has been reached on whether PI is natural or an artifact and on what appropriate approach and surrogate biomarkers should be used for PI investigation [7].

In TEAM, Cold (寒) and Heat (熱), two opposing patterns, are used to describe the nature of a phenomenon (e.g., illness or body type). Theoretically, the Heat pattern refers to acute symptoms such as severe, reddish, and swollen pain accompanied by other heat-related signs such as aversion to hot temperature, hot sensation in the limbs and abdomen, facial flushing, thirst, and dark urine, whereas the Cold pattern includes cold-type symptoms (e.g., chronic and dull pain) and signs (e.g., cold temperature, cold sensation in the limbs and the abdomen, pale face, no thirst, and clear urine) [8]. Few attempts have been made to interpret the nature of the Cold-Heat patterns using the concepts of modern physiology. Nonetheless, it has been shown that the Cold-Heat pattern in rheumatoid arthritis is related to C-reactive protein [3] and has a genetic basis [5]. Recent evidence suggests that an imbalance in the endocrine renin-angiotensin system may result in Cold and Heat patterns [9]. Li et al. [6] found that the Cold pattern is associated with increased leptin levels, indicative of a reduction in energy metabolism, whereas the Heat pattern is accompanied by hyperactive immune regulation. However, these findings were derived from observations of patients suffering from particular diseases (e.g., rheumatoid arthritis or gastritis) with a complex array of symptoms and signs. Thus, in these cases the Cold and Heat patterns may have been interacting with other pathogens, such as Dampness (*濕邪*) and Wind (*風邪*). Unfortunately, there has been no study on the impact of Cold and Heat patterns in healthy subjects.

In modern physiology, the perception of coldness/hotness belongs to the scope of thermal comfort. In a particular environment, humans are not uniform in their judgment of the thermal condition. Although there is no difference in body core and skin temperature, some individuals feel colder than others in an air-conditioned environment [10, 11], whereas some tend to have a lower warm sensation threshold [12]. Thermal comfort also varies between body regions. Nakamura et al. [13], using stimulated hot and cold conditions, found that humans prefer a cooler face and warmer abdomen, respectively. They also reported that the distal region (e.g., limbs and extremities) is less sensitive to changes in thermal pleasantness than the trunk [14]. Interestingly, the phenomenon of cold limbs is reported to have a genetic basis [15]. However, these findings were based on laboratory experiments using a relatively small sample size.

According to the TEAM theory, general feelings of coldness/hotness in the hands, legs, and abdomen are three important dimensions in the questionnaire used to determine the Cold-Heat patterns [8]. Although these perceptions are subjective, they may reflex the capability of temperature adaptation in the context of thermoregulation. Individual variations in temperature acclimatization are influenced by various factors, including age, sex, body composition, and illnesses [16, 17]. However, no research has been performed on the contribution of these factors to the Cold-Heat patterns.

The present population-based study aimed to investigate factors (e.g., anthropometric indices, thermal regulation parameters, and metabolic and obesity-related biomarkers) potentially involved in variations in the subjective perception of coldness/hotness and to examine whether these factors contribute to regional thermal comfort in the hands, legs, and abdomen (Cold-Heat sensation). Our results shed light on the essence of the Cold-Heat patterns in TEAM as well as thermal sensation in modern physiology.

## 2. Methods

### 2.1. Study Setting and Population

The present cross-sectional study was conducted as part of a project aimed at developing the Korean Constitutional Multicenter Bank [18] at Asan Medical Center, Seoul, Republic of Korea, from 2009 to 2015. A total of 978 apparently healthy volunteers aged from 20 to 69 years who had not suffered from any chronic disease and had no history of hospitalization in the previous 5 years were recruited through advertisements. Eligible participants underwent body composition analysis, blood work-up (including metabolic and obesity-related biomarkers), and an oral glucose tolerance test (OGTT). Those who had missing data on any blood sampling items or the questionnaire (*n* = 182) were excluded. We also excluded those who had evidence of high blood pressure (systolic blood pressure ≥ 140 mmHg and/or diastolic blood pressure ≥ 90 mmHg) and/or diabetes (fasting plasma glucose ≥ 126 mg/dL and/or plasma glucose at 120 min of OGTT ≥ 200 mg/dL) (*n* = 62) [19]. Finally, 734 participants (369 men and 365 women) were included in the analysis. The study was approved by the Asan Medical Center ethics committee and informed consent was obtained from all participants.

### 2.2. Cold-Heat Sensation Score

Participants were asked to complete a self-report questionnaire containing three questions “*Generally, are your hands cold or warm?,*” “*Generally, are your legs cold or warm?,*” and “*Generally, is your abdomen cold or warm?*” by selecting the answer that is closest to their personality including “Cold,” “Medium,” or “Warm.”. Then, each dimension was scored as −1, 0, or +1, respectively. The three regional Cold-Heat sensation scores were then used in the subsequent analysis. The Cronbach alpha value for internal consistency was 0.83 for the hand and leg scores but 0.72 for the hand, leg, and abdomen scores.

To explore the Cold-Heat sensation in general by gender, the overall Cold-Heat balance score was calculated as the summation of the hand, leg, and abdomen scores and it ranges from −3 to +3. The values below zero indicated a trend toward the “Cold” type, whereas the positive values indicated a trend toward the “Heat” type.

### 2.3. Blood Assay

The baseline blood assay included liver enzymes (aspartate aminotransferase (AST) and alanine aminotransferase (ALT)), thyroid hormones (free T3, free T4, and TSH), stress hormones (epinephrine, norepinephrine (NE), and cortisol), and lipid profile (total cholesterol, triglycerides, HDL cholesterol, and LDL cholesterol). An OGTT was performed according to the standard protocol and as described previously [20]. In brief, participants fasted for at least 12 hours before the experiment and were asked to consume 75 g glucose (Glu-orange; McNulty Pharmaceutical, Seoul, Korea). Plasma concentrations of glucose (GLU.0, GLU.30, GLU.60, GLU.90, GLU.120, and GLU.180) and insulin (Insulin.0, Insulin.30, Insulin.60, Insulin.90, Insulin.120, and Insulin.180) were measured at the baseline and at 30, 60, 90, 120, and 180 minutes during the OGTT.

### 2.4. Body Composition

Body composition, including body fat, water, protein, and mineral mass, was analyzed using bioimpedance analysis with the InBody 720 (Biospace, Seoul, Korea). The percentage of body fat was the proportion of body fat mass over body weight. Body weight and height were measured using a digital scale. Body mass index (BMI) was calculated as weight in kilograms over height in meters squared. Based on the body composition results, body heat capacity (HC), the energy in kilocalories needed to raise the temperature of the body by 1°C, was calculated using specific HC coefficients as 1, 0.507, 0.229, and 0.201 for water, fat, protein, and mineral mass, respectively [21].

### 2.5. Thermoregulation-Related Parameters

Participants were asked to refrain from the consumption of any stimulants (smoking, alcohol, and coffee), heavy exercise, and eating for at least 12 hours before the test. Oxygen consumption (VO_2_) and carbon dioxide production (VCO_2_) at rest were measured using indirect calorimetry with canopy mode (Vmax®Encore; SensorMedics, VIASYS Healthcare, Yorba Linda, CA). Measurements were taken over 20 minutes while participants were lying supine and awake on a bed and wearing a sealed hood connected to the system. Flow and gas calibrations were performed following the guideline released by the producer. Resting energy expenditure (REE) was then calculated by a modified Weir equation [22]. REE measurements were conducted at an ambient temperature of 23 ± 1°C and humidity of 60%. Temperature increment load at resting state (*T*_load_) in degree Celsius per hour was calculated as REE divided by HC [23]. REE and *T*_load_ were used as determinants of heat production (*H*_prod_) and heat load (*H*_load_), respectively, under resting conditions.

### 2.6. Data Analysis

Data were analyzed using R software version 3.2.2 for Windows 7. Differences in the proportions of responses to Cold-Heat patterns in each gender were analyzed using the chi-square test for independence. Differences in continuous variables across Cold-Heat sensation score groups were analyzed by analysis of variance (ANOVA). To adjust for age, gender, and BMI, an analysis of covariance (ANCOVA) was used. Analyses were performed separately for the hand, leg, and abdomen scores.

## 3. Results

In total, 369 men and 365 women were included in the final analysis. Men were older and heavier but had less body fat than women. HC, REE, and *T*_load_ were higher in men (*p* < 0.001). To raise the body temperature 1°C, women needed almost 2SDs less energy (12 kcal/°C) than men. Although the difference in the REE between men and women was relatively high (>1SD), the difference in *T*_load_ was modest (almost 0.25 SD) (Table 1). The differences in HC, REE, and *T*_load_ according to gender remained after adjusting for age and BMI (Table 2).

**Figure 1:**
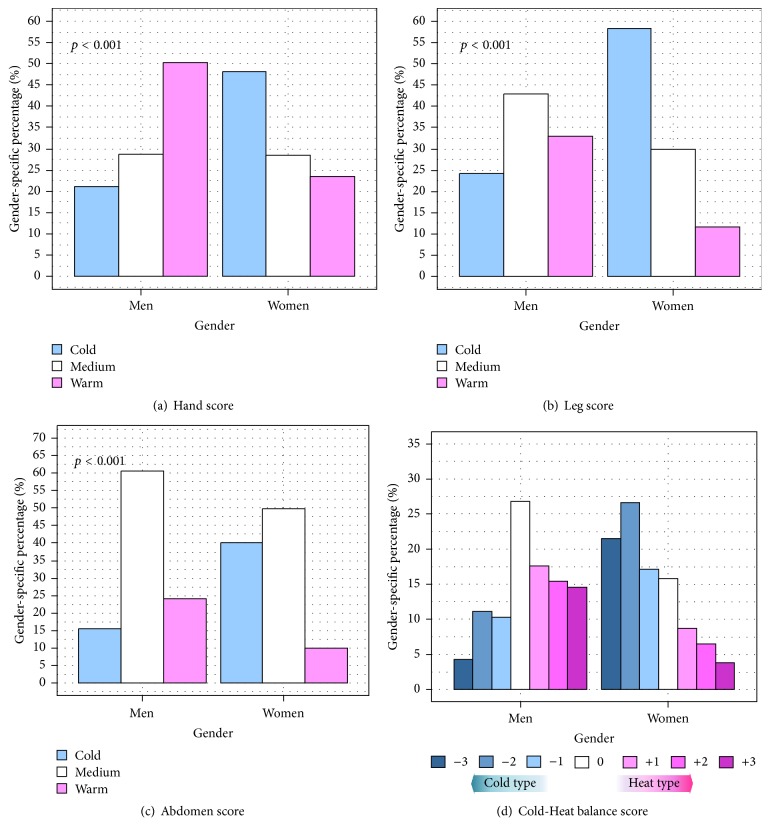
Distribution of response to Cold-Heat questionnaire by gender. *p* values were calculated by chi-square test for independence.

The response to the Cold-Heat questionnaire was not uniform in each gender, particularly for the sensation of coldness/hotness in the hands and legs. Half of the men reported that they had warm hands in general, whereas around 20% of them chose the opposite answer (Figure 1(a)); this trend was reversed in women. The proportion of “Cold” responders for the leg and abdomen were 58.6% and 40.3% of women and 23.8% and 15.4% of men, respectively (Figures 1(b) and 1(c)). The tendency for women to be likely to rate their body sensation as the Cold type was more clearly revealed in the analysis of the overall Cold-Heat balance score: 65.2% of women versus 25.5% of men were considered to be of the “Cold” type, whereas 19.2% of women versus 47.7% of men were considered to be of the “Heat” type (Figure 1(d)).

### 3.1. Coldness/Hotness versus Anthropometric and Thermoregulation Parameters

In comparison with “Cold” respondents, “Medium” and “Warm” respondents had a higher weight, body fat mass, percentage of body fat, HC, and REE in analyses of the hand, leg, and abdomen scores. However, these differences disappeared after adjusting for age, gender, and BMI. “Warm” and “Medium” respondents had higher *T*_load_ than “Cold” respondents in the analysis of the abdomen score only. However, these differences also disappeared after adjusting for age, gender, and BMI (Table 3).

ANCOVA with adjustment for age, gender, and body weight for the hand score showed that “Warm” and “Medium” respondents had a 0.74 and 0.36 kg/m^2^ higher BMI than “Cold” respondents, respectively, whereas the difference between “Warm” and “Medium” respondents was 0.38 kg/m^2^. The same analysis of the leg score indicated that “Warm” and “Medium” respondents had a 0.52 and 0.31 kg/m^2^ higher BMI than “Cold” respondents, respectively, whereas there was no consistent difference in the BMI in the analysis of the abdomen score (Table 3).

### 3.2. Sensation of Coldness/Hotness versus Biomarkers

The biomarkers screened in this study could be categorized as metabolic determinants (epinephrine, NE, free T3, free T4, TSH, and cortisol), as well as liver function (AST, ALT) and lipid profile (total cholesterol, triglycerides, HDL cholesterol, and LDL cholesterol), and as being related to glucose metabolism (glucose and insulin during OGTT). In general, it appeared that “Warm” and “Medium” respondents to the hand, leg, and abdomen scores had higher AST, ALT, NE, and free T3 levels than “Cold” respondents. However, after adjusting for age, gender, and BMI, these differences disappeared, except for that of NE. For the hand score, “Warm” and “Medium” respondents had a NE level of around 57 pg/mL higher than that of “Cold” respondents (*p* < 0.05). For the leg score, the difference in NE after adjustment for confounding factors between “Warm” and “Cold” respondents was 50 pg/mL (*p* < 0.05). No similar consistent difference was found for analysis of the abdomen score (Table 4).

**Figure 2:**
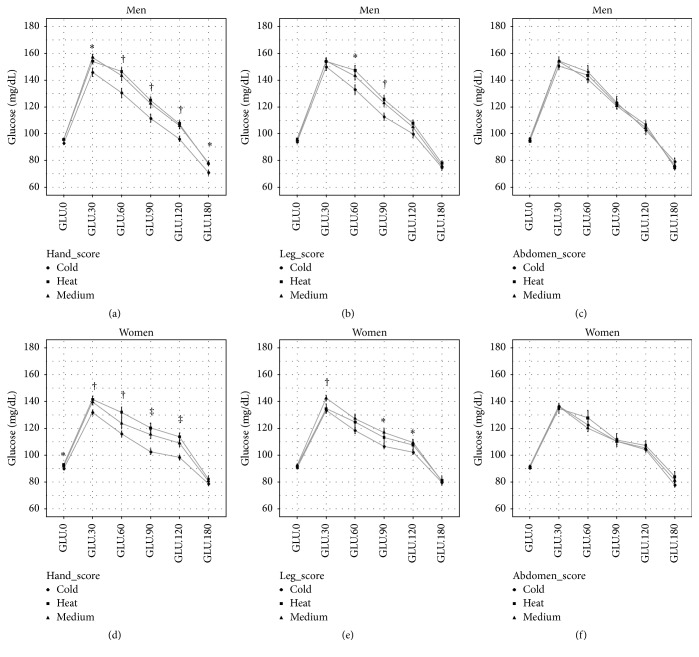
Plasma glucose overtime during OGTT across Cold-Heat sensation score groups. Data are mean and SE. ^*∗*^ *p* < 0.05; ^†^ *p* < 0.01; ^‡^ *p* < 0.001.

**Figure 3:**
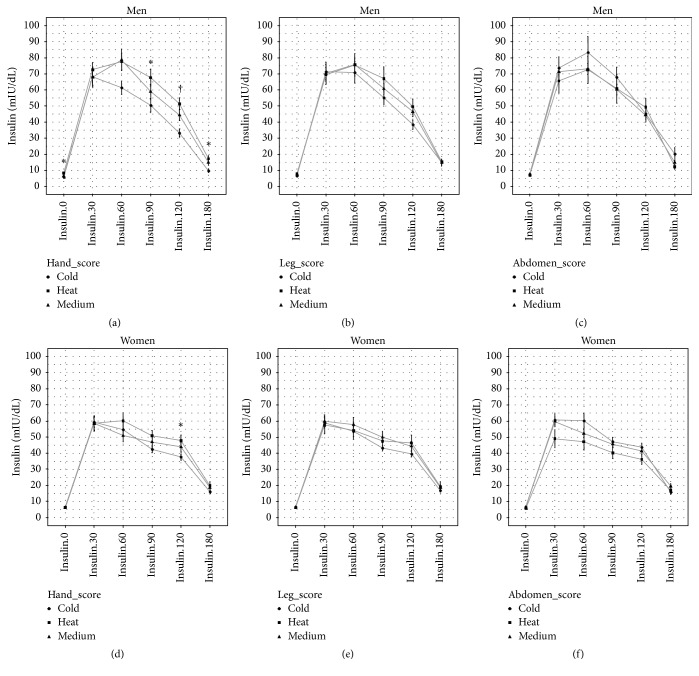
Plasma insulin overtime during OGTT across Cold-Heat sensation score groups. Data are mean and SE. ^*∗*^ *p* < 0.05; ^†^ *p* < 0.01.

Although there was no difference in GLU.0 and Insulin.0 between “Warm,” “Medium,” and “Cold” respondents to the hand and leg scores, the magnitude of the increment in glucose and insulin during OGTT of the “Warm” and “Medium” respondents was larger than that of the “Cold” respondents. This pattern was significant in the analysis of the hand score, whereas no difference was found in the analysis of the abdomen score (Figures 2 and 3). After adjusting for age, gender, and BMI, glucose (GLU.30, GLU.60, GLU.90, GLU.120, and GLU.180) and insulin (Insulin.120 and Insulin.180) were higher for “Warm” and “Medium” respondents to the hand score than “Cold” respondents; in addition, this trend also appeared at several time points for glucose measurement during OGTT in the analysis of the leg score. No consistent difference was found in the analysis of the abdomen score (Table 5).

## 4. Discussion

The main findings of our present study were as follows: (1) women are more likely to perceive their regional thermal sensation to be cooler than warmer, (2) the perception of coldness/hotness of the hands and legs is BMI related but not that of the abdomen, and (3) the perception of coldness/hotness for the limbs, particularly the hands, is associated with plasma NE and glucose metabolism, independently of BMI.

Sex differences in thermal sensation and comfort have been investigated worldwide. Results from a recent review study reported that women find it substantially more difficult to adapt to a change in the thermal environment than men [24]. In a study of Swiss individuals aged from 20 to 40 years, women were 4.5 times more likely to complain of cold hands and legs than men [25]. As reported by Nagashima et al. [10], nearly one-third of Japanese women reported having unusual cold extremities; few men made the same report. In the present study, with an age range from 20 to 68 years, the same trend in gender-specific thermal comfort/discomfort was seen not only in the hand and leg dimensions, but also in the abdomen dimension. When we integrated these three dimensions, the tendency became stronger, with women 2.6 times more likely to be the “Cold” type than men and men 2.5 times more likely to be the “Warm” type than women. Evidence indicates that women have a higher core temperature (*T*_core_) than men under all conditions (e.g., during rest, exercise, and sleep) [26], whereas the blood flow in hands was higher in men at rest than in women [27]. Furthermore, the skin temperature reduction due to cold exposure [28] and heat loss (*H*_loss_) via sweating [29] was lower in women than in men. We also found that the basal *H*_prod_, *H*_load_, REE, and *T*_load_ were lower in women, independently of BMI. In terms of thermoregulation, we can assume that the lower *H*_prod_ and *H*_load_ due to female-specific anthropometric characteristics (e.g., small body size and low fat-free mass) are offset by the restricted heat dissipation process that may induce a lower peripheral heat flow and consequently a hyperdissatisfaction to thermal change, particularly to cold conditions, in women.

It has been reported previously that an inclination toward perception of cold extremities was related to BMI in a Swiss population [11]. On a similar note, we also found that BMI was the most crucial anthropometric factor influencing the Cold-Heat sensation, particularly hand and leg scores. Adjustment for age, gender, and body weight revealed that individuals with warmer hands and legs were more likely to have a higher BMI, whereas no specific effect was found for thermal perception in the abdomen. Although obese people consume more energy and have larger *H*_prod_, no difference has been found in the *T*_core_ between the different BMI groups [26, 30]. To retain the inner thermal balance condition, obese people therefore need to boost their heat dissipation to control their large temperature load. Under resting conditions, almost all *H*_loss_ is conducted via constitutive heat exchange, including radiation, convection, and respiration [23], and this process seems to be region dependent. Savastano et al. [30] indicated that, under thermoneutral conditions, obese people had a 5°C higher fingernail temperature (*T*_finger_) and 1°C lower abdomen skin temperature (*T*_abdomen_) than normal weight individuals. Because no difference in *T*_core_ was found between our BMI groups, a relatively small variation in the abdominal core-to-skin temperature gradient between obese and lean individuals may not be sufficient to induce a substantial difference in the perception of abdominal thermal comfort. In addition, a thick cutaneous adipose layer, particularly in the trunk and abdominal regions, functions as a thermoinsulation layer that inhibits the proximal core-to-skin heat exchange in higher BMI individuals. Therefore, to compensate for greater *H*_prod_, obese people need to increase *H*_loss_ via distal dry heat exchange, such as that from the hands and legs. Experiments involving mild cold exposure also revealed that the skin temperature reduced more at the extremities than at the abdomen, chest scapula, and lower back in lean subjects than in obese individuals [31]. Interestingly, in line with that finding, in the current study, the abdomen score was not associated with BMI, an indicator of obesity.

Augmented *H*_loss_ from the extremities in people with a high BMI may be related to some physiological metabolic defect associated with this phenotype. Previously, the Cold-Heat pattern in TEAM was associated with activity and the neuro-endocrine-immune network [9]. Our present study is the first to attempt to screen the relationship between various cold exposure-related biomarkers (e.g., thyroid hormone, cortisol, vasoactive hormone, lipid profile, and glucose metabolism test) and thermal comfort under thermoneutral conditions. A consistent difference was found only in NE and the glucose metabolism test across the respondents for the hand and leg scores. The fact that those who had warmer hands and legs were more likely to have a lower plasma NE and a higher increase in plasma glucose and insulin during OGTT underlined the sympathetic nervous system basis of the thermal sensation. Increased NE during cold exposure generates more heat for body warming [32]. However, at a given cold stimulation, a higher secretion of this hormone was found in lean compared with obese individuals [31]. Some may argue that the lower sympathetic nervous system activity in “Warm” respondents is influenced by their higher BMI, but adjustment for this confounding factor confirmed the independent impact of NE on thermal comfort. Because *T*_core_ is identical in unusually cold hand subjects and normal ones [10], we can assume that the increment in NE in “Cold” respondents is solely a peripheral response to offset their chronic lower peripheral thermal state to reduce the heat loss. This assumption needs to be investigated further.

Interestingly, the dynamic change in glucose and insulin during OGTT was higher in those who reported warm hands than in “Cold” respondents, even though participants were diabetes and hypertension-free. This phenomenon was also independent of BMI. It has been shown that insulin secretion increases capillary blood flow in muscle [33], which might partially explain the warmer sensation in those who had a hyperactive response to OGTT. Because the phenomenon was seen only for the hands and not for the legs and abdomen, a region-dependent effect of glucose metabolism on thermal comfort could be a future study subject.

In TEAM, the Cold-Heat pattern includes heterogeneous dimensions such as perception of coldness/hotness, symptom appearance (e.g., acute and severe versus chronic and dull), face color, and quality of secretion/urination and stool [8], in which Cold-Heat sensation plays an important role in every examination of Cold and Heat patterns. Generally, those who generally perceive cold sensation at limbs and abdomen are clinically diagnosed to be the “Cold” type and/or may be suffered from Cold syndrome [2, 8]. This method is similar for determining the “Heat” type. However, in the context of modern physiology, these dimensions evidently do not refer to a unique mechanism. Because the perception of coldness/hotness is a key indicator of Cold-Heat patterns, influencing factors of body thermal sensation such as gender, BMI, NE, and glucose metabolism should be considered when performing Cold-Heat pattern identification (*寒熱*辨證). In pathological progression of a certain illness, it has been unclear why someone develops Cold pattern while others develop Heat pattern. Further study should focus on whether those who generally belongs to Cold type are more likely to suffer from Cold pattern illnesses and whether influencing factors of Cold-Heat sensation interact significantly with this progression. Furthermore, physiological basis of Cold-Heat sensation within the context of Cold-Heat patterns in TEAM is regionally different. The feeling of coldness/hotness in the hands and legs seems to concur with the concept of thermal comfort, whereas that of the abdomen may be based on another physiological basis (e.g., digestive function).

The findings of our present study should be interpreted in accordance with its strengths and limitations. This is the first attempt to explore the dimension-specific basis of the Cold-Heat sensation, a key indicator of Cold-Heat pattern concept in TEAM, to enroll a relatively large sample size with various screening parameters such as body composition, thermoregulation, physiological, and metabolic parameters. Because our analysis was designed as a cross-sectional study, the causal effects of BMI, gender, NE, and glucose metabolism on the sensation of coldness/hotness in the hands and legs were not confirmed. The questionnaire for each dimension was set up as a three-point scale and might not provide a concrete conclusion. Furthermore, the perception of coldness/hotness for the whole body was not used, limiting the applicability of our findings. Finally, because Cold-Heat sensation reflects one aspect of Cold-Heat patters in TEAM, the findings should not be extended to the concept of Cold (寒) and Heat (熱) in general.

In conclusion, our data suggest that gender and BMI are determinants of the perception of coldness/hotness in the hands and legs, whereas such a relationship may not be appropriate for the perception of the Cold-Heat sensation in the abdomen. Some biological markers such as NE and the dynamic change in glucose and insulin may contribute to the sensation of coldness/hotness in the extremities. These influencing factors of regional Cold-Heat sensation should be taken into account in Cold-Heat pattern identification (*寒熱*辨證).

## Figures and Tables

**Table 1:** Anthropometric indices and thermoregulation linked parameters by gender.

Variable	Men	Women	*p* value
Number of participants (*n*)	369	365	
Age (yr)	34.0 (10.5)	36.7 (11.6)	0.001
Weight (kg)	71.3 (9.6)	56.2 (6.8)	<0.001
BMI (kg/m^2^)	23.8 (2.7)	22.0 (2.7)	<0.001
Body fat mass (kg)	14.6 (5.6)	16.7 (4.8)	<0.001
Percent body fat (%)	20.1 (5.7)	29.2 (5.9)	<0.001
Heat capacity (kcal/°C)	53.2 (6.4)	40.4 (4.4)	<0.001
REE (kcal/d)	1716.8 (343.9)	1242.2 (198.1)	<0.001
*T*_load_ (°C/hr)	1.35 (0.23)	1.29 (0.19)	<0.001

Data are presented as mean (SD) or actual number of participants. *p* values were calculated by Student *t*-test. BMI, body mass index; REE, resting energy expenditure; *T*_load_, resting temperature load.

**Table 2:** Differences in thermoregulation parameters between men and women.

Variable	Men	Women	*p* value
Heat capacity (kcal/°C)	ref	−9.6 (−10.1 to −9.1)	<0.001
REE (kcal/d)	ref	−385.9 (−425.0 to −346.8)	<0.001
*T*_load_ (°C/hr)	ref	−0.07 (−0.10 to −0.04)	<0.001

Data are presented as regression coefficient and 95% confident interval calculated by analysis of covariance. Men were employed as the reference (ref). All analyses were adjusted for age and BMI.

**Table 3:** Difference in anthropometric characteristic and thermoregulation parameters across Cold-Heat sensation score groups.

	ANOVA				ANCOVA		
	Cold	Medium	Warm	*p*	Medium versus Cold	Warm versus Cold	Warm versus Medium
*Hand score*							
*n*	253	210	271				
Weight (kg)	58.5 (9.3)	63.3 (9.8)	69.1 (11.5)	<0.001	−0.33	−0.41	−0.08
BMI (kg/m^2^)^¶^	21.5 (2.3)	22.8 (2.5)	24.2 (3.0)	<0.001	0.36^†^	0.74^‡^	0.38^†^
Body fat mass (kg)	14.4 (4.3)	15.4 (5.1)	17.0 (6.0)	<0.001	−0.17	−0.07	0.10
Percent body fat (%)	24.7 (6.9)	24.5 (7.6)	24.6 (7.7)	0.948	0.01	0.19	0.18
Warm capacity (kcal/°C)	42.9 (7.2)	46.5 (7.5)	50.7 (8.5)	<0.001	−0.20	−0.30	−0.09
REE (kcal/d)	1369.1 (313.2)	1467.8 (339.3)	1595.2 (401.9)	<0.001	−5.75	−25.14	−19.39
*T*_load_ (°C/hr)	1.33 (0.19)	1.31 (0.21)	1.31 (0.24)	0.61	0.00	−0.01	−0.01
*Leg score*							
*n*	302	267	165				
Weight (kg)	58.8 (9.0)	65.9 (10.7)	69.4 (12.1)	<0.001	0.03	−0.14	−0.18
BMI (kg/m^2^)^¶^	21.8 (2.4)	23.4 (2.7)	24.1 (3.2)	<0.001	0.31^*∗*^	0.52^‡^	0.22
Body fat mass (kg)	15.0 (4.6)	15.9 (5.4)	16.3 (6.2)	0.025	−0.36	−0.42	−0.06
Percent body fat (%)	25.7 (6.9)	24.1 (7.7)	23.4 (7.5)	0.002	−0.73^*∗*^	−0.69	0.04
Warm capacity (kcal/°C)	43.0 (6.9)	48.4 (8.0)	51.1 (8.8)	<0.001	0.15	0.03	−0.13
REE (kcal/d)	1362.3 (308.5)	1531.6 (355.7)	1615.6 (419.8)	<0.001	7.04	−16.43	−23.47
*T*_load_ (°C/hr)	1.32 (0.19)	1.32 (0.21)	1.32 (0.26)	0.984	0.00	−0.01	−0.02
*Abdomen score*							
*n*	204	404	126				
Weight (kg)	60.9 (10.1)	64.1 (11.0)	67.4 (12.6)	<0.001	−0.10	−0.75	−0.64
BMI (kg/m^2^)^¶^	22.6 (2.9)	22.8 (2.8)	23.6 (3.0)	0.004	−0.06	0.28	0.34^*∗*^
Body fat mass (kg)	16.6 (5.4)	15.1 (5.0)	15.8 (5.9)	0.003	−0.43	−0.46	−0.03
Percent body fat (%)	27.3 (7.2)	23.6 (7.1)	23.4 (7.4)	<0.001	−0.64	−0.40	0.24
Warm capacity (kcal/°C)	44.1 (7.4)	47.2 (8.3)	49.7 (9.2)	<0.001	0.06	−0.46	−0.52
REE (kcal/d)	1349.8 (295.8)	1512.8 (365.4)	1590.4 (420.8)	<0.001	40.51	22.22	−18.29
*T*_load_ (°C/hr)	1.28 (0.19)	1.33 (0.21)	1.33 (0.26)	0.006	0.03	0.03	0.00

For ANOVA analysis, data are presented as mean (SD).

For ANCOVA, data are presented as mean difference. “Medium versus Cold” and “Warm versus Cold” and “Cold” responses were employed as the reference; “Warm versus Medium” and “Medium” responses were employed as the reference. All analyses were adjusted for age, gender, and BMI. ^¶^ANCOVA analysis for BMI was adjusted from age, gender, and body weight. Significant level: ^*∗*^ *p* < 0.05; ^†^ *p* < 0.01; ^‡^ *p* < 0.001.

**Table 4:** Differences in biomarkers across Cold-Heat sensation score groups.

	ANOVA analysis				ANCOVA analysis		
	Cold	Medium	Warm	*p*	Medium versus Cold	Warm versus Cold	Warm versus Medium
*Hand score*							
*n*	253	210	271				
AST (U/L)	17.4 (5.8)	19.1 (6.2)	21.1 (17.3)	0.001	0.31	1.52	1.22
ALT (U/L)	12.5 (7.1)	15.5 (10.1)	19.6 (20.8)	<0.001	−0.18	1.35	1.53
Epinephrine (pg/mL)	50.7 (69.5)	52.1 (54.6)	55.9 (94.2)	0.73	−2.72	7.48	10.2
Norepinephrine (pg/mL)	295.1 (279.3)	258.2 (186.8)	242.1 (188.0)	0.02	−57.6^*∗*^	−58.3^*∗*^	−0.68
Free T3 (ng/mL)	1.3 (0.2)	1.3 (0.3)	1.4 (0.2)	0.001	−0.03	−0.01	0.02
Free T4 (*μ*g/mL)	8.1 (1.9)	8.2 (2.2)	8.4 (2.1)	0.25	0.13	0.11	−0.02
TSH (*μ*IU/mL)	2.3 (1.5)	2.3 (1.4)	2.3 (1.5)	0.83	−0.04	0.05	0.09
Cortisol (*μ*g/dL)	11.4 (4.9)	11.7 (5.1)	11.7 (4.8)	0.78	0.26	0.27	0.01
Total cholesterol (mg/dL)	179.6 (28.8)	182.6 (31.7)	183.7 (31.4)	0.28	−2.55	−3.12	−0.57
Triglycerides (mg/dL)	84.7 (43.9)	100.6 (87.5)	114.2 (82.8)	<0.001	0.71	3.07	2.36
HDL cholesterol (mg/dL)	59.5 (17.4)	55.9 (15.8)	53.3 (18.0)	<0.001	−0.81	0.27	1.07
LDL cholesterol (mg/dL)	100.3 (31.7)	104.1 (32.9)	106.7 (34.5)	0.08	−2.12	0.79	2.92
*Leg score*							
*n*	302	267	165				
AST (U/L)	17.6 (6.1)	20.5 (15.9)	20.3 (10.7)	0.007	0.97	0.23	−0.74
ALT (U/L)	13.0 (8.2)	16.6 (11.6)	20.3 (24.1)	<0.001	−0.49	1.77	2.26
Epinephrine (pg/mL)	51.8 (68.7)	52.4 (82.3)	56.2 (78.7)	0.83	−3.04	5.92	8.97
Norepinephrine (pg/mL)	282.3 (256.1)	267.1 (213.6)	230.0 (169.1)	0.05	−30.0	−50.2^*∗*^	−20.2
Free T3 (ng/mL)	1.3 (0.2)	1.3 (0.3)	1.4 (0.2)	0.007	−0.03	−0.02	0.00
Free T4 (*μ*g/mL)	8.1 (2.1)	8.2 (2.0)	8.4 (2.1)	0.28	0.07	−0.01	−0.09
TSH (*μ*IU/mL)	2.3 (1.5)	2.2 (1.4)	2.4 (1.5)	0.66	−0.07	0.08	0.15
Cortisol (*μ*g/dL)	11.3 (5.0)	11.5 (5.0)	12.4 (4.8)	0.07	0.15	0.94	0.79
Total cholesterol (mg/dL)	179.7 (28.7)	184.2 (31.8)	182.6 (32.1)	0.21	−0.83	−1.80	−0.98
Triglycerides (mg/dL)	87.5 (46.7)	104.6 (72.6)	116.1 (107.5)	<0.001	−1.74	4.23	5.96
HDL cholesterol (mg/dL)	59.0 (17.5)	54.7 (16.3)	53.3 (18.1)	0.001	0.02	1.17	1.15
LDL cholesterol (mg/dL)	100.1 (31.3)	107.4 (34.3)	104.4 (34.3)	0.03	2.04	1.17	−0.88
*Abdomen score*							
*n*	204	404	126				
AST (U/L)	17.7 (5.6)	19.7 (13.8)	20.4 (11.0)	0.07	1.13	0.98	−0.15
ALT (U/L)	13.9 (9.0)	15.8 (12.0)	20.0 (25.3)	0.001	0.47	2.68	2.22
Epinephrine (pg/mL)	45.2 (52.5)	59.1 (92.4)	46.1 (40.6)	0.05	13.6^*∗*^	1.58	−11.9
Norepinephrine (pg/mL)	276.4 (279.5)	268.6 (212.6)	235.1 (145.7)	0.23	−2.29	−31.03	−28.74
Free T3 (ng/mL)	1.3 (0.2)	1.3 (0.2)	1.4 (0.2)	0.05	−0.02	−0.03	−0.02
Free T4 (*μ*g/mL)	8.0 (2.0)	8.4 (2.1)	8.1 (2.0)	0.05	0.12	−0.30	−0.42
TSH (*μ*IU/mL)	2.5 (1.7)	2.2 (1.3)	2.3 (1.5)	0.038	−0.30^*∗*^	−0.13	0.17
Cortisol (*μ*g/dL)	10.9 (5.1)	11.7 (4.7)	12.4 (5.2)	0.016	0.45	1.07	0.61
Total cholesterol (mg/dL)	183.1 (30.8)	181.4 (30.9)	181.9 (29.8)	0.80	−0.09	−1.17	−1.08
Triglycerides (mg/dL)	100.6 (75.3)	99.0 (74.1)	103.0 (74.0)	0.86	−7.62	−12.93	−5.31
HDL cholesterol (mg/dL)	57.5 (18.0)	56.0 (16.6)	54.7 (18.7)	0.34	1.64	2.86	1.22
LDL cholesterol (mg/dL)	104.3 (30.5)	103.5 (33.4)	103.6 (36.8)	0.95	1.12	0.14	−0.98

For ANOVA analysis, data are presented as mean (SD).

For ANCOVA, data are presented as mean difference. “Medium versus Cold” and “Warm vs Cold” and “Cold” responses were employed as the reference; “Warm versus Medium” and “Medium” responses were employed as the reference. All analyses were adjusted for age, gender, and BMI. Significant level: ^*∗*^ *p* < 0.05.

**Table 5:** Differences in plasma glucose and insulin during OGTT across Cold-Heat sensation score groups adjusted for age, gender, and BMI.

	Hand score			Leg score			Abdomen score		
	Cold	Medium	Warm	Cold	Medium	Warm	Cold	Medium	Warm
GLU.0 (mg/dL)	ref	1.23	1.53	ref	0.66	1.11	ref	−0.75	−1.54
GLU.30 (mg/dL)	ref	7.22^†^	7.14^†^	ref	4.62^*∗*^	2.87	ref	0.6	−2.49
GLU.60 (mg/dL)	ref	5.83	10.99^†^	ref	4.49	7.79^*∗*^	ref	−1.66	1.63
GLU.90 (mg/dL)	ref	8.19^†^	10.08^‡^	ref	5.61^*∗*^	6.50^*∗*^	ref	0.6	0.31
GLU.120 (mg/dL)	ref	6.94^†^	8.76^‡^	ref	2.11	3.8	ref	2.78	3.9
GLU.180 (mg/dL)	ref	3.69	3.82^*∗*^	ref	0.46	1.83	ref	1.59	0.81

Insulin.0 (mIU/dL)	ref	−0.68	−0.33	ref	−0.29	−0.72	ref	−0.22	−1.24^*∗*^
Insulin.30 (mIU/dL)	ref	−1.02	−4.77	ref	−1.3	−7.33	ref	−1.53	−11.3
Insulin.60 (mIU/dL)	ref	3.68	0.51	ref	1.58	−4.32	ref	−8.39	−13.28^*∗*^
Insulin.90 (mIU/dL)	ref	3.85	3.79	ref	2.59	1.6	ref	−3.28	−7.83
Insulin.120 (mIU/dL)	ref	6.26^*∗*^	6.42^*∗*^	ref	3.09	2.17	ref	−1.62	−2.4
Insulin.180 (mIU/dL)	ref	4.12^*∗*^	4.10^*∗*^	ref	0.94	−0.3	ref	0.45	−3.27

Data are mean difference compared with cold group.

ANCOVA analysis adjusted for age, gender, and BMI. Significant level: ^*∗*^ *p* < 0.05; ^†^ *p* < 0.01; ^‡^ *p* < 0.001.
